# Changes in the 24 h Rhythmicity of Liver PPARs and Peroxisomal Markers When Feeding Is Restricted to Two Daytime Hours

**DOI:** 10.1155/2011/261584

**Published:** 2011-04-05

**Authors:** Julieta B. Rivera-Zavala, Adrián Báez-Ruiz, Mauricio Díaz-Muñoz

**Affiliations:** Departamento de Neurobiología Celular y Molecular, Instituto de Neurobiología, Campus UNAM-Juriquilla, Querétaro 76230, QRO, Mexico

## Abstract

Restricted feeding (RF) during daytime is associated with anticipatory activity before feeding, marked hyperphagia after mealtime, adjustments in hepatic metabolism, and the expression of a food-entrained oscillator (FEO). 24 h rhythmicity of liver PPAR*α*, *β*, and *γ*, peroxisomal markers (PMP70, AOX, and catalase), and free fatty acids (FFAs) during RF was evaluated. The effect of fasting-refeeding was also studied. Results showed (1) higher levels of FFA before feeding, (2) a shift of PPAR*α* and PPAR*γ* before and of PPAR*β* peaks after feeding, (3) an increase in peroxisomal markers, (4) a shift of PMP70 and AOX peaks before feeding, and of maximal catalase activity in the dark period, (5) changes in the fasting-refeeding response, and (6) high correlations (>0.9) of serum corticosterone with PPAR*α* and PPAR*γ* and of PMP70 with PPAR*β*. In conclusion, 24 h rhythmicity of FFA, liver PPARs, and peroxisomal markers are biochemical adaptations associated with daytime RF and FEO expression.

## 1. Introduction

Circadian rhythms are ~24 h cycles that allow a finely tuned adaptation of metabolic, physiological, and behavioral responses to environmental cues [1]. These daily fluctuations are part of a timing system constituted by a hierarchical assembly of multiple endogenous oscillators. Among these oscillators, a major pacemaker synchronized by the alternation of light and dark periods is localized in the suprachiasmatic nucleus (SCN) of the hypothalamus [2]. However, the timing system is differentially modulated when food access is restricted to period of 2–4 h daily for consecutive days [3]. Animals manifest, prior to feeding, a behavior known as food anticipatory activity (FAA), which encompasses an increase in locomotion and body temperature, elevated levels of circulating corticosterone, and the activation of digestive enzymes [4, 5]. Because the circadian responses to restricted feeding schedules persist even when the SCN is ablated, the existence of an alternative oscillator entrained by food (FEO) has been postulated [6]. 

Alternation between feeding and fasting involves a rhythmic progression in the assimilation and mobilization of nutrients. These physiological activities are under endocrine and neural control, and in the liver, they also require the concerted induction and repression of defined anabolic and catabolic pathways. It has become evident that these biochemical adaptations are reciprocally associated with components of the molecular clock [7]. The mechanism underlying this circadian clock exist in virtually every mammal cell and includes a positive (Per1-3/Cry1, 2) and a negative feedback loop (Bmal1/Clock/NPAS2) [8]. Moreover, the molecular clock involves other feedback loops (nutrient-sensing elements such as ROR*α*, Rev-erb*α*, and PPAR*α*) and posttranslational modifications (phosphorylation, ubiquitination, and acetylation) to sustain the circadian rhythmicity [9]. Hence, the timing system and metabolic networks in the liver influence each other by controlling the redox state, sirtuin activity, energy charge, intracellular calcium dynamics, and so forth [10].

Restricted feeding (RF) and the concomitant FEO expression promote changes in the metabolic handling of energetic substrates by the liver and adipose tissue FFA [11, 12]. For example, lipolytic release of free fatty acids (FFAs) and production of ketone bodies are increased [13], whereas in the liver, the levels of triacylglycerols are reduced and the glycogen is only partially hydrolyzed [14]. These findings strongly suggest that the protocol of RF for 2 h in daytime is accompanied by an enhancement of FFA, and later, by increased oxidation of these molecules within the liver. Hence, one of the aims of this project was to further explore the regulation of hepatic lipid metabolism in RF by testing the response of the peroxisome proliferator-activated receptors (PPARs) and, consequently, the participation of peroxisomal activity during this feeding protocol.

PPARs are members of the nuclear receptor superfamily that is involved in the daily metabolic regulation of nutrients. They act as transcription factors in conjunction with the retinoid X receptor (RXR). Three subtypes have been identified PPAR*α*, PPAR*β*, and PPAR*γ*, and all of them are expressed in the liver. PPAR*α* is the most abundant in hepatic tissue; it regulates genes controlling the number and function of peroxisomes and is important in the fasting response [15]. PPAR*β* acts as a hepatic sensor of circulating FFA and promotes coordination between glucose and fatty acid metabolism, thereby modulating energy homeostasis [16, 17]. PPAR*γ* regulates triacylglyceride metabolism, controlling hepatic steatosis and protecting against insulin resistance [18]. Studies of mRNA expression of the liver PPARs demonstrated that all of them show robust 24 h rhythmicity [19].

Peroxisomes are specialized organelles involved in lipid synthesis (formation of bile acids and plasmalogens) and degradation (oxidation of very-long-chain fatty acids) [20]. They act in coordination with mitochondria to complete the process of *β*-oxidation in cases of a large supply of dietary lipids or during fasting conditions [21]. Peroxisomes are also enriched in enzymes such as catalase that catabolizes H_2_O_2_, which is a by-product of several peroxisomal enzymes. Liver is one of the major organs expressing meal-dependent peroxisomal activity.

Hence, to further understand the diurnal adaptations associated with the protocol of RF in FFA handling, PPAR signaling, and liver peroxisomal activity, the present study aimed to characterize the 24 h rhythmicity of (1) circulating FFA, (2) hepatic content of PPAR*α*,*β*, and *γ*, and (3) markers of peroxisome number (peroxisome membrane protein of 70 kDa, PMP70), peroxisomal *β*-oxidation (acyl-CoA oxidase, AOX), and peroxisomal H_2_O_2_-handling activity (catalase) in rats maintained under RF (food access from 12:00 to 14:00 h).

## 2. Methods

### 2.1. Animals and Housing

Male Wistar rats weighing 180 ± 20 g were maintained under constant conditions of 12:12 h LD cycle (lights on 08:00 h) and temperature (22 ± 1°C). Rats were kept in groups of 4 in transparent acrylic cages (40 × 50 × 20 cm) with free access to Purina Chow and water except during food restriction or fasting conditions. Experimental procedures were conducted in accordance with our Institutional Guide for Care and Use of Experimental Animals (Universidad Autónoma de México) and conformed to international ethical standards previously recommended [22].

### 2.2. Experimental Design

Control and experimental groups were similar to those reported previously [23]. To determine daily and food-entrained rhythmicity, rats were randomly assigned to one of the following feeding conditions for 3 weeks:

1. control animals fed *ad libitum* with free access to food and water throughout the 24 h period,
2. experimental group with RF, food availability from 12:00 to 14:00 h,
3. at the end of the feeding protocol, different subgroups of animals was sacrificed at 3 h intervals over a 24 h period, starting at 08:00 h.

In addition, 2 additional groups were included to compare the fasting and subsequent refeeding response in the RF group:

1. animals fed *ad libitum* were maintained with free food access for 3 weeks; on the last day, food was removed at 14:00 h, and they were sacrificed (at 11:00 h) after 21 and 45 h (~1 and 2 days) of deprivation;
2. a second group of rats was similarly deprived of food for 21 and 45 h, then refed for 2 h (from 12:00 to 14:00) and sacrificed at 14:00 h before tissue sampling.

### 2.3. Liver and Blood Sampling

Rats were beheaded with a guillotine-like instrument. Three to four mL of trunk blood was collected in 10 mL silicone-coated test tubes containing a clot-activator gel (Vacutainer), and then centrifuged at 3,000 rpm for 15 min to obtain blood serum. The liver was removed (*≈*5 g) and immediately placed in an ice-cold isolation medium (1 : 10 wt/vol) containing 250 mM sucrose, 0.1% BSA (fatty acid free) and 0.5 mM EGTA (pH 7.4). The tissue was homogenized with a Teflon homogenizer (40 rpm for 10 s). Hepatic protein was measured by the Lowry method [24]. 

### 2.4. Determination of Free Fatty Acids

The FFA were quantified with a commercial kit (no. 612-100, BioVision) by the conversion of long-chain free fatty acids to their CoA derivatives that are measured at 570 nm. 

### 2.5. Determination of PPARs and Peroxisomal Markers

PPAR*α*, PPAR*β*, PPAR*γ*, PMP70, and AOX were measured in liver homogenates of each individual rat and the 8 times tested elapsing the 24 h period by Western blotting. Equal amounts of protein (50 *μ*g) were mixed with 2X Laemmli sample buffer (Bio-Rad), separated in a 12% polyacrylamide gel, electroblotted onto a nitrocellulose membrane, and then incubated overnight with the primary antibodies against the 3 PPARs (Santa Cruz), PMP70 (Invitrogen), AOX, and *β*-actin (Abcam) at 1 : 500 dilution. The next day, membranes were washed 3 times (2% NaCl and 0.1% Tween) and incubated for 2 h with alkaline phosphatase- (AP-) conjugated secondary antibodies: rabbit antigoat (INVITROGEN) for the 3 PPARs, donkey antirabbit (Santa Cruz) for PMP70 and AOX, and donkey antimouse (Santa Cruz) for *β*-actin, at 1 : 5000 dilution. Bands were visualized using the AP-conjugate substrate (Bio-Rad) according to the manufacturer's instructions. Western blots were done in duplicate, one to detect the corresponding PPAR protein and the other to detect the presence of actin which was considered as a control of loading. The determinations from all rats were expressed as the ratio of densitometric signals of PPARs/actin. Results were plotted with the mean and the SEM of the numerical values of the 4 rats tested in each temporal point. 

### 2.6. Catalase Activity

50 *μ*g of total liver protein extract was incubated with phosphate buffer (50 mM KH_2_PO_4_, pH 7.0) in a final volume of 3 mL for 4-5 min to establish a stable background. To initiate the enzymatic reaction, 5 *μ*L of hydrogen peroxide (~30%) was added. The catalase activity was recorded in the initial 3 min as a reduction in absorbance at 240 nm [25].

### 2.7. Data Analysis

Data were classified by group and time and are presented as mean ± standard error of the mean (SEM). Data were compared using a two-way ANOVA for independent measures, with a factor for group (2 levels) and a factor for time (8 levels). In order to determine significant time effects for each daily sampling profile, a one-way ANOVA was performed for individual groups. The one- and two-way ANOVAs were followed by a Tukey post hoc test with the significance threshold set at *P* < .05. The daily profiles of PPARs and peroxisomal markers as well as corticosterone levels were examined to determine the Pearson's Correlation coefficient. Statistical analysis was performed with the program Statistica, version 4.5 (StatSoft, Inc.).

## 3. Results

### 3.1. The 24 h Rhythmicity of Serum Free Fatty Acids Is Modified by RF

**Figure 1:**
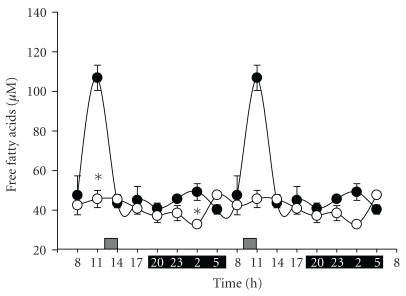
Effect of restricted feeding on daily variations of free fatty acids. Double-plotted representation of temporal profiles of long-chain free fatty acids measured in serum over two daily cycles. Control group fed *ad libitum* (●) and group with RF (∘). Data are presented as mean ± SEM of 3 rats at each time point. Black bar depicts the dark period (from 20:00 to 08:00 h), and the gray bar indicates the time of food access (from 12:00 to 14:00 h). Time points with a significant difference between *ad libitum* and RF groups are highlighted with an asterisk (*) (Tukey post hoc test, *P* < .05).

FFA are energetic molecules that are mobilized during fasting or intense exercise.  Figure 1 shows the time course of FFA for groups fed *ad libitum* or with a restricted feeding (RF) schedule. Control rats showed discrete changes in FFA, with higher values (~25%) in the light period (resting time) than in the dark period (meal time). In contrast, RF elicited an exceptional response at 11:00 h, prior to food access and during FAA, the peak value increased by ~140% relative to the average value of the other time points. Another significant difference was observed at 02:00 h, when the RF group showed a ~40% elevation.

### 3.2. The 24 h Rhythmicity of Hepatic PPARs Is Modified by RF

**Figure 2:**
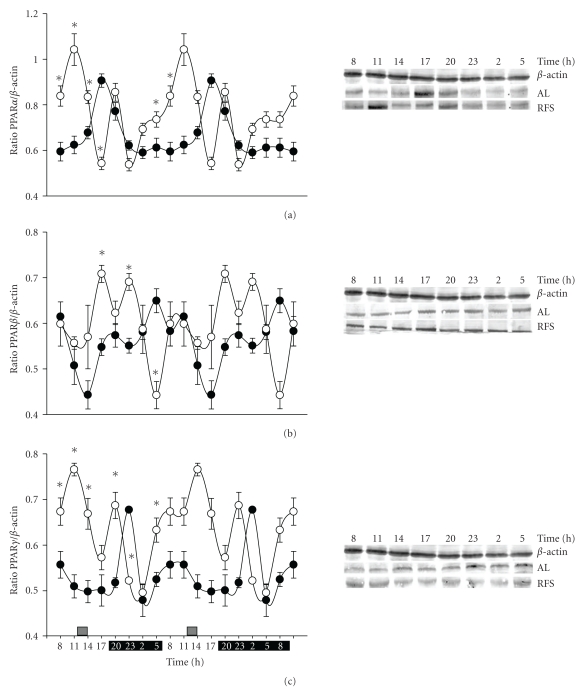
Effect of restricted feeding on daily expression of liver PPARs. Double-plotted representation of diurnal expression in liver homogenates (50 *μ*g of protein) of (a) PPAR*α*, (b) PPAR*β*, and (c) PPAR*γ.* Control group fed *ad libitum* (AL, ●) and group with RF (∘). Results were normalized to the content of *β*-actin in parallel determinations to adjust for loading differences. Representative western blot experiments are displayed at the right of each graph. Data are presented as mean ± SEM of 4 rats from each time point. Black bar depicts the dark period (from 20:00 to 08:00 h), and the gray bar indicates the time of food access (from 12:00 to 14:00 h). Time points with a significant difference between *ad libitum* and RF groups are marked with an asterisk (*) (Tukey post hoc test, *P* < .05).

In the liver, PPARs are important transcription factors that regulate the metabolism of nutrients, but mainly fatty acid oxidation and lipid storage. The control group fed *ad libitum* exhibited a robust rhythmicity in PPAR*α* and PPAR*γ* levels, with a peak at the transition between the light and dark periods (17:00–20:00 h for PPAR*α*) and during the first part of the dark period (23:00 h for PPAR*γ*) (Figures 2(a) and 2(c)). These peaks represent increases of 30–50% above average values. PPAR*β* showed a different type of rhythmicity with a significant valley detected in the middle of the light period (14:00 h) (Figure 2(b)). The level of PPAR*β* at this time was ~50% lower than the average value. In contrast, the 24 h rhythmic fluctuations of the 3 hepatic PPARs in the experimental group with RF revealed significant changes, both in temporal patterns and in amplitude (Figures 2(a)–2(c)): diurnal variations of PPAR*α* and PPAR*γ* were similar; they showed a significant peak prior to food access (11:00 h), a second smaller peak at the beginning of the dark period (20:00 h), and a valley in the dark period (23:00–02:00 h) (Figures 2(a) and 2(c)). PPAR*β* levels exhibited two peaks 17:00 and 23:00 h and a marked valley at the end of the dark period (05:00 h) (Figure 2(b)).

The 24 h average content of the 3 PPARs tended to increase in the rats under restricted feeding schedules (13% PPAR*α*, 7% PPAR*β*, and 18% PPAR*γ*), primarily during the light period, when food was available (Table 1). The amplitude of the variations displayed by the PPAR*γ* in the group entrained by food was 2 times higher than that shown by the rats fed *ad libitum* (Table 1).

### 3.3. Effect of Feeding Conditions (Fasting versus Refeeding) on the Level of Hepatic PPARs (Western Blot)

**Figure 3:**
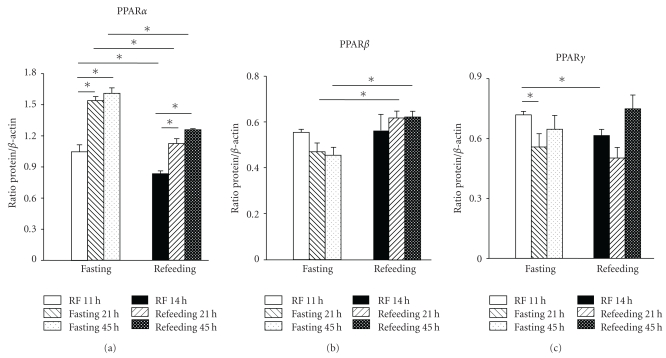
Effect of fasting and refeeding on liver PPARs. Liver homogenates (50 *μ*g of protein) were analyzed for the expression of PPAR*α*, PPAR*β*, and PPAR*γ.* Results were normalized to the content of *β*-actin in parallel determinations to adjust for loading differences. Fasted groups: RF at 11:00 h, 21 h and 45 h without food, and refeeding groups: RF at 14:00 h, 21 h + 2 h of food access, and 45 h + 2 h of food access. Data are presented as mean ± SEM of 4 rats from each temporal point. Each group of fasted and refed groups was compared by one-way ANOVA (Tukey post hoc test, *P* < .05); significant differences between fasting versus refed groups were detected by the Student's *t*-test- (*P* < .05).

PPAR*α* was sensitive to the conditions of fasting/refeeding: 2 h of feeding decreased the level of this transcription factor by 25–30% compared to the level reached after 21 h and 45 h of fasting. The same pattern was shown in the RF groups, since the PPAR*α* level decreased ~18% after feeding (comparing 11:00 h with 14:00 h); however, PPAR*α* in rats under RF was significantly lower (~30%) than in fasting/refeeding groups (Figure 3(a)). The response of PPAR*β* to feeding was different: refeeding after 21 and 45 h of fasting increased the level of PPAR*β* by ~25%; in contrast, rats with RF did not show any change between fasting (11:00 h) and feeding (14:00 h). Feeding conditions had a minimal effect on the expression of hepatic PPAR*γ*: 21 and 45 h of fasting with subsequent 2 h refeeding did not change the level of this factor; rats with RF showed higher levels of PPAR*γ* before (11:00 h) than after mealtime (14:00 h). In addition, after a single 21 h fast, the PPAR*γ* level was lower than in the fasting group with RF (at 11:00 h).

### 3.4. The 24 h Rhythmicity of Hepatic Peroxisomal Markers Is Modified by RF

**Figure 4:**
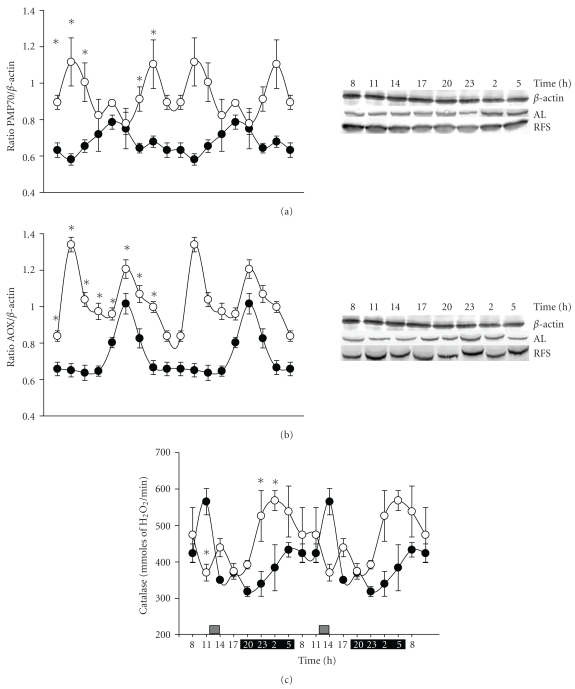
Effect of restricted feeding on daily expression of liver peroxisomal markers. Double-plotted representation of diurnal expression in liver homogenates (50 *μ*g of protein) of (a) PMP70, (b) AOX, and (c) catalase activity*.* Control group fed *ad libitum* (AL, ●) and group with RF (∘). Results for PMP70 and AOX were normalized to the content of *β*-actin determined in parallel determinations to adjust for loading differences. Representative western blots for PMP 70 and AOX are displayed on the right. Data are presented as mean ± SEM of 4 rats from each time point. Black bar depicts the dark period (from 20:00 to 08:00 h), and the gray bar indicates the time of food access (from 12:00 to 14:00 h). Time points with significant difference between *ad libitum* and RF groups are depicted with an asterisk (*) (Tukey post hoc test, *P* < .05).

Figure 4 shows the diurnal fluctuations of 3 peroxisomal markers: PMP70, a membrane transporter for acyl-CoA derivatives into peroxisomes; AOX, which initiates the peroxisomal *β*-oxidative cycle; and catalase, an H_2_O_2_-metabolizing enzyme. The 3 markers showed a 24 h rhythm in the control group fed *ad libitum*, with the peaks at 20:00 h (PMP70, panel (a)), 23:00 h (AOX, panel (b)), and 11:00 h (catalase, panel (c)). Food restriction promoted significant changes in the rhythmic profile of all these peroxisomal markers: PMP70 and AOX levels showed rhythms with 2 peaks (one during FAA at 11:00 h and the other in the dark period), and their average levels were significantly higher than controls (panels (a) and (b)); the peak of catalase activity shifted, with the highest value occurring during the dark period (panel (c)). 

The average values of PMP70, AOX, and catalase activity in the groups with RF were clearly greater than those of the group fed *ad libitum* (38% for PMP70, 44% for AOX, and 19% for catalase activity), strongly suggesting an increase of liver peroxisomal activity in the rats synchronized by food. The amplitude of the PMP70 fluctuations in the group under RF increased by 50% compared to the control rats with free food access. PMP70 and particularly AOX were clearly elevated during the light period. In contrast, catalase activity was higher in the dark period (Table 1). 

### 3.5. Effect of Feeding Conditions (Fasting versus Refeeding) on Hepatic Peroxisomal Markers

**Figure 5:**
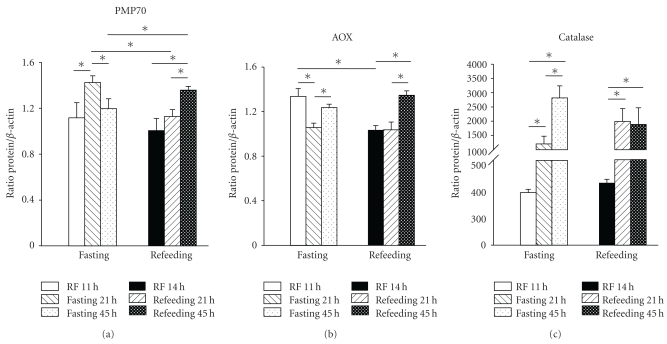
Effect of fasting and refeeding on liver peroxisomal markers. Liver homogenates (50 *μ*g of protein) were analyzed for the expression of PMP70, AOX, and catalase activity*.* Results for PMP70 and AOX were normalized to the content of *β*-actin in parallel determinations to adjust for loading differences (or as above). Fasted groups: RF at 11:00 h, 21 h, and 45 h without food, and refeeding groups: RF at 14:00 h, 21 h + 2 h of food access, and 45 h + 2 h of food access. Data are presented as mean ± SEM of 4 rats from each time point. The fasted and refed groups were compared by one-way ANOVA (Tukey post hoc test, *P* < .05); significant differences for fasting versus refed groups were detected by the Student's *t*-test (*P* < .05).

Peroxisomal activity is sensitive to the feeding state: it has been reported that a 24 h fast enhances the number of liver peroxisomes [26]. Indeed, our results in rats fed *ad libitum* confirmed this information: levels of PMP70 were higher in 21 h fasted rats than in the refeeding group (~28) (Figure 5(a)). This response was not observed in the groups with 2 days of fasting followed by refeeding. Rats with RF did not show any change due to feeding conditions in the PMP70 level and showed a significant increase (~25%) in AOX protein after mealtime (Figures 5(a) and 5(b)). In contrast, 21 and 45 h of fasting promoted a dramatic increase in hepatic catalase activity (~210 and 400%, resp.) in comparison to the RF rats prior to meal access (11:00 h) (Figure 5(c)). 

### 3.6. Correlation Analysis among PPARs, Peroxisomal Markers, and Corticosterone

**Figure 6:**
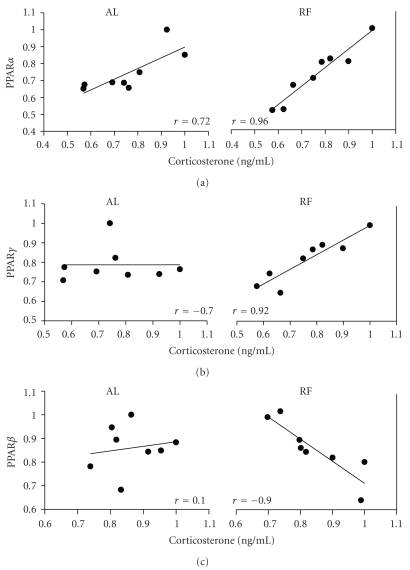
Correlation between liver PPARs, serum corticosterone, and the liver peroxisomal marker PMP70 in rats under restricted feeding. Control group fed *ad libitum* (left panels) and group with RF (right panels). Correlations were analyzed by plotting the corresponding daily values for each PPAR (Figure 2), circulating corticosterone (taken from [26]), and PMP70 (Figure 4(a)). *r* = correlation coefficient.

To determine if the changes promoted by the restricted feeding in the time patterns of liver PPARs coincided with the fluctuations of peroxisomal markers and serum corticosterone [26], a correlation analysis was performed (Figure 6). It has been proposed that glucocorticoids act as modulators of PPAR expression [17]. Of all the correlations tested, just 3 comparisons had a high correlation coefficient (*r* of at least 0.90) in the group with RF: PPAR*α* and corticosterone showed a moderate correlation (*r* = 0.72) in the group fed *ad libitum*, but in the rats with RF, the correlation was much higher (*r* = 0.96) (panel (a)). PPAR*γ* showed no correlation with circulating corticosterone in rats with free access to the food; however, in rats with RF these parameters showed a high correlation (*r* = 0.92) (panel (b)). Similarly, PPAR*β* exhibited a high but negative correlation with PMP70 (*r* = −0.90) in the group with restricted feeding, but there was no correlation in the rats fed *ad libitum* (panel (c)). 

## 4. Discussion

### 4.1. PPARs and the Molecular Clock

PPARs play a strategic role in the bidirectional control between metabolic networks and the molecular clock [27]. This concept is well documented for peripheral oscillators with high metabolic activity and capacity to process nutrients, such as the liver. For example, (1) treatment with the PPAR*α* ligand bezafibrate affected the phase of clock genes such as Per2, Bmal1, and Rev-erb*α* in liver and other organs [28]; (2) there is a regulatory feedback loop between PPAR*α* and Bmal1 [29]; (3) miR-122, a hepatocyte-specific microRNA is under the control of Rev-erb*α*, whereas PPAR*β* and coactivators of PPAR*α* are targets of miR-122 for circadian regulation [30]; (4) clock participates in the modulation of lipid metabolism by the circadian transactivation of PPAR*α* [31].

### 4.2. PPARs, Peroxisomal Markers, and Feeding Conditions

Previous reports have documented that mRNA expression of PPARs is modulated differentially by food access: during a 12 h fast, PPAR*α* and *γ* levels were 50 and 10% higher, respectively, than those after refeeding, whereas PPAR*β* increased 60% during the meal after a similar fast [32]. Furthermore, daily expression profiles of genes encoding these nuclear receptors in rats fed* ad libitum* showed 24 h rhythmicity with peaks at the beginning of the dark phase [33]. These results agree with the western blot data of the present study (Figure 3). Upregulation of PPAR*α* expression is associated with the activation of hepatic fatty acid oxidation and is considered to be a critical event in the adaptative response to the fasting state [15, 34]. In our protocol of restricted feeding, prior to meal time (11:00 h), FFA availability [11] coincides with high levels of corticosterone, a peak in the concentration of the clock protein Per1 [26], and the larger peaks of PPAR*α* and PPAR*γ* (Figure 2)*.* Interestingly, liver mRNA levels for glucocorticoid receptors showed no changes during the fasting condition [35]. Our results were consistent with this pattern, with augmented PPAR*β* after feeding in control (during the dark period) and food-restricted groups (during light period) (Figure 1). Hence, during RF, liver PPARs shifted phases and increased the amplitude of their rhythms, but they responded to feeding conditions as previously reported.

Diurnal fluctuations of the peroxisomal proteins PMP70 and AOX are regulated by PPAR*α* under the influence of the clock proteins clock and Bmal1 [36, 37]. The results shown in Figure 4 for *ad libitum* and RF groups are consistent with this type of regulation. PMP70 and AOX also showed a second peak at the beginning of the dark phase in the rats under restricted feeding schedules; this could be interpreted as a remaining influence of the metabolic response driven by the light-dark cycle (Figures 4(a) and 4(b)).

A 24 h fast is a well-known condition that increases the peroxisomal activity [38]. This response is usually linked to an enhanced lipolysis and FFA availability. RF promoted a large increase in FFA levels prior to food access (11:00 h, Figure 1) concomitant with an elevation of ketone bodies [11]. This condition makes some of the ligands that are most effective in promoting PPAR-induced responses [39] available. All 3 peroxisomal markers tested, PMP70, AOX, and catalase, were present at higher average levels over the 24 h period in the group with RF (Figure 3). However, the 24 h pattern of PMP70 and AOX (higher values during the light period) differed from the one shown by catalase (higher values during the darkness period). One possible explanation for this variation is the existence of peroxisomal subpopulations or a differential regulation in the enzymatic outline of the hepatic peroxisomes in function of nutrient processing.

### 4.3. Metabolic Adaptations to Daytime Food Restriction

The PPAR*γ* coactivator 1*α* (PGC-1*α*) is part of the transcriptional mechanism by which PPAR*α* influences intermediary metabolism in the liver, including the induction of the enzymes involved in mitochondrial fatty acid oxidation, such as carnitine palmitoyl transferase 1*α* (CPT-1*α*) [40]. Unpublished data obtained by our group using DNA microarray experiments suggest that a significant increase in the mRNA for PGC-1*α* occurs in the rats under restricted feeding at 11:00 h (during FAA) and at 14:00 h (after food intake). Corticosterone, which is elevated at the same time as PPAR*α* (at 11:00 h, during FAA), positively regulates the PPAR*α* gene at the transcriptional level [41], suggesting that both are part of a concerted response in the group with RF. 

PPAR*γ* contributes to the clearance of blood glucose and the accumulation of triacylglycerides in the liver since its ablation prevented fatty liver [18]. It has also been reported that agonists of PPAR*γ* inhibit the activation of hepatic stellate cells [42]. Our results showed similar daily fluctuations of PPAR*γ* and PPAR*α* under both *ad libitum* feeding and RF conditions (a single peak in the control group and a complex pattern with a significant elevation just before meal time in the RF group, Figure 2). The parallel patterns of these two transcription factors suggest a coordinated transcriptional response and putative synchronized actions during FAA, mainly elevated production of ketone bodies [13], and the control of triacylglyceride levels within the liver [14].

The function of PPAR*β* and PPAR*α* in the liver is coordinated during fatty acid oxidation [43]. Indeed, the transcription of PPAR*β* is associated with food intake and mediates the expression of lipogenic genes induced by glucose and insulin. In addition, an interesting relationship between PPAR*β* and the inhibitory control of gluconeogenesis has been postulated, since PPAR*β* and the lipogenic factor SREBP-1c showed similar expression patterns during fasting and refeeding [17, 29]. Unpublished results from our laboratory indicate an inverse correlation in *ad libitum* and food restricted groups between the activity of the gluconeogenic marker phosphoenol pyruvate carboxykinase (PEPCK) and the presence of PPAR*β*, supporting the notion that PPAR*β* could be playing a similar role in our experimental protocol. PPAR*β* also acts as a hepatic sensor of the level of circulating FFA [14].

### 4.4. Correlations

The relationships between PPAR*α* and corticosterone are well documented [37] and are confirmed by our data in the *ad libitum* and restricted feeding groups (Figure 6(a)), indicating that PPAR*α* and corticosterone correlated but were independent of FEO expression. In contrast, the high correlations observed between PPAR*γ* and circulating corticosterone and between PPAR*β* and the peroxisomal marker PMP70 were exclusively observed in the groups under RF (Figures 6(b) and 6(c)). These results strongly suggest that under restricted feeding schedules and the associated FEO expression, the liver could show novel physiological and metabolic responses involving cell signaling and peroxisomal regulation.

In conclusion, the present study provides evidence that the PPARs and peroxisomal markers vary over the course of the 24 h cycle depending on the feeding protocol. The adjustments promoted by the RF schedule strongly suggest a global adaptation in the metabolic handling of nutrients in the liver. This metabolic fine-tuning includes not only changes in the timing of the peaks but also important variations in the amplitude of their daily rhythms, suggesting that a rheostat-like physiological regulator is associated with food restriction and most probably with the expression of the FEO. 

## Figures and Tables

**Table 1:** Comparison between groups fed *ad libitum* (control) and under restricted feeding schedules (RFS) of average values and the ratio of data corresponding to the light and dark periods.

	FFA		PPAR*α*		PPAR*β*		PPAR*γ*		PMP70		AOX		CATALASE	
	CTRL	RF	CTRL	RF	CTRL	RF	CTRL	RF	CTRL	RF	CTRL	RF	CTRL	RF
Light mean	44	61	0.70	0.81	0.53	0.61	0.51	0.67	0.65	0.96	0.65	1.05	427.70	414.61
Dark mean	39	44	0.65	0.71	0.59	0.59	0.55	0.58	0.71	0.92	0.83	1.06	369.08	506.29
L/D	1.1	1.4	1.08	1.14	0.90	1.03	0.47	0.57	0.92	1.04	0.78	0.99	1.16	0.82
% change L/D		↑27		↑6		↑14		↑21		↑13		↑27		↓29

Averages were taken from data in Figures 1 and 3. Up arrow indicates increase and down arrow indicates decrease in the percentage of change between values of the light and dark periods.
